# Noise-Tolerant Force Calculations in Density Functional Theory: A Surface Integral Approach for Wavelet-Based Methods

**DOI:** 10.1021/acs.jpca.4c06708

**Published:** 2025-01-27

**Authors:** Moritz Gubler, Jonas A. Finkler, Stig Rune Jensen, Stefan Goedecker, Luca Frediani

**Affiliations:** † Department of Physics, University of Basel, Klingelbergstrasse 82, CH-4056 Basel, Switzerland; ‡ Hylleraas Centre, Department of Chemistry, UiT the Arctic University of Norway, Tromso̷ N-9037, Norway; § Department of Chemistry and Bioscience, Aalborg University, Fredrik Bajers Vej 7H, 9220 Aalborg Øst, Denmark

## Abstract

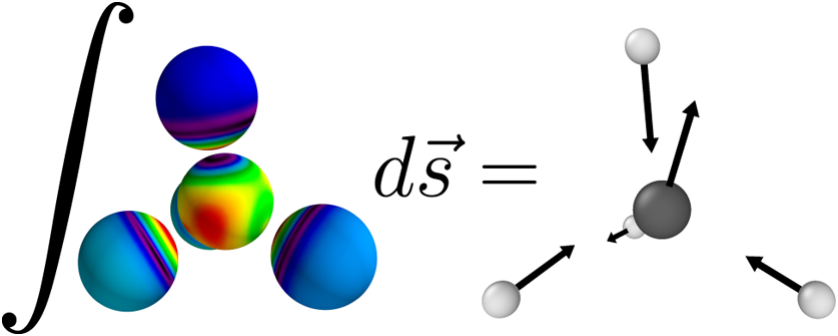

We introduce a method for computing quantum mechanical forces through surface integrals over the stress tensor within the framework of the density functional theory. This approach avoids the inaccuracies of traditional force calculations using the Hellmann–Feynman theorem when applied to multiresolution wavelet representations of orbitals. By integrating the quantum mechanical stress tensor over surfaces that enclose individual nuclei, we achieve highly accurate forces that exhibit superior consistency with the potential energy surface. Extensive benchmarks show that surface integrals over the stress tensor offer a robust and reliable alternative to the direct use of the Hellmann–Feynman theorem for force computations in DFT with discontinuous basis sets, particularly in cases where wavelet-based methods are employed. In addition, we integrate this approach with machine learning techniques, demonstrating that the forces obtained through surface integrals are sufficiently accurate to be used as training data for machine-learned potentials. This stands in contrast to forces calculated using the Hellmann–Feynman theorem, which do not offer this level of accuracy.

## 1 Introduction

Density functional theory (DFT)^1,2^ has become a cornerstone in the computational study of molecular and condensed matter systems due to its balance of accuracy and computational efficiency. Central to the practical application of DFT is the accurate calculation of forces acting on nuclei, which is essential for geometry optimizations,^3−5^ molecular dynamics simulations,^6^ and various other simulations. The Hellmann–Feynman theorem^7^ provides a direct route to force calculations by relating the forces to expectation values of gradients of the Hamiltonian with respect to nuclear positions, thus avoiding the calculation of derivatives of molecular orbitals.

In DFT, the choice of basis sets is crucial for the accuracy and efficiency of calculations. Common basis sets like Gaussian-type orbitals (GTOs)^8^ offer computational efficiency and ease of integration but cannot fully eliminate basis set errors due to their inability to accurately represent nuclear cusps and their nonsystematic nature. Numerical atomic orbitals (NAOs)^9−12^ are tuned to resemble physical orbitals and can for example represent nuclear cusps better than GTOs. The dependence of GTOs and NAOs on atomic positions complicates force calculations, often rendering the Hellmann–Feynman theorem inapplicable and necessitating the computation of Pulay forces.^13,14^

In contrast, systematic basis sets like wavelets and plane waves do not suffer from this issue, making them more efficient for accurate force calculations. Wavelets^15−17^ allow for adaptive resolution and efficient handling of both smooth and sharply varying functions. Plane waves can also be used as basis sets in bulk materials. However, their inability to accurately represent the cusps in the orbitals at the position of the nuclei necessitates the use of pseudopotentials.^18,19^ Recent advancements in all electron pseudopotentials^20,21^ have enabled all-electron calculations within a plane wave framework.

Each of these basis sets has its own advantages and is suited to different types of problems in electronic structure calculations.^22^

In this paper, we will focus on multiwavelets (MWs)^23^ as basis sets which are used in the codes M-A-D-N-E-S-S(16) and MRChem.^17^ Multiwavelets, in the formulation of Alpert,^24^ correspond in essence to using polynomials on adaptive grids in such a way that the error is kept rigorously under control.^23^ Coupled with the separated representation of the main Green functions^25^ (Poisson and Helmholtz kernels) they enabled Hartree–Fock (HF)^26^ and DFT^27^ calculations with this method to achieve very high precision.^28^ In 20 years the model has matured from a niche for precise benchmarks on small molecules^27,29^ to a robust tool for production calculations for energies^28,30,31^ and properties^32−34^ including both correlated methods^35,36^ and relativistic Hamiltonians.^37−39^ Despite these developments, a robust and reliable way to compute gradients (an indispensable tool for any computational chemistry practitioner) has been lacking: the current implementation based on the Hellmann–Feynman theorem requires very high precision to yield acceptable results and is therefore of limited applicability.^40^ With this development we aim to provide a method to compute geometrical derivatives at enhanced precision, which will make multiwavelet methods appealing for quantum chemistry applications.

When multiresolution wavelets are used to represent the Kohn–Sham orbitals, the singularity of the 1/*r* electron–nucleus potential complicates integral evaluations which can be solved by using a smooth form of the nuclear potential.^40^ The introduction of this finite, smooth nuclear potential enables the use of the Hellmann–Feynman theorem to compute the forces acting on the nuclei.

However, this approach may encounter noise due to the rapidly changing nature of the approximate nuclear potential and its derivative. To address these issues, we propose to calculate forces by evaluating surface integrals over the quantum mechanical stress tensor.^41,42^ This approach mitigates numerical problems inherent in traditional methods by taking the integration surfaces far away from the nuclei, thus reducing the spatial variation of the orbitals. It is therefore particularly well-suited for use with wavelet-based DFT methods. By integrating the stress tensor over a carefully chosen surface that encloses each nucleus, forces can be computed from regions far from the nuclear cusp in the orbitals, thereby avoiding potential numerical issues caused by these cusps. We believe that stochastic methods like self-averaging Kohn–Sham DFT^43^ and techniques using discontinuous Galerkin orbitals^44,45^ could also benefit from this approach due to its ability to avoid nuclear cusps.

In this paper, we detail the theoretical foundations of our method, starting with a review of the Hellmann–Feynman theorem and its limitations. We then introduce the concept of the stress tensor in the context of DFT and describe how surface integrals over this tensor can be used to compute forces. Our method is then benchmarked against the traditional approach using a variety of molecular systems, demonstrating its superior accuracy.

## 2 Theoretical Background

Unless otherwise specified, atomic units (a.u.) are used throughout this study.

### 2.1 Hellmann–Feynman Theorem

In the generalized gradient approximation of the Kohn–Sham density functional theory,^1,2^ the total energy has the following form:
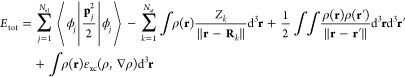
1

The single particle wave functions |ϕ_*k*_⟩ are eigenstates of the Kohn–Sham Hamiltonian
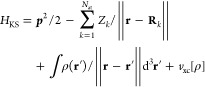
 that fullfill a single-particle Schrödinger equation *H*_KS_ϕ_*k*_ = ε_*k*_ϕ_*k*_. Forces acting on the nucleus *k* are the negative gradient of eq 1 with respect to the position of the nucleus **R**_*k*_.

Derivatives of quantum mechanical expectation values can be calculated using the Hellmann–Feynman theorem^7^ which states that . The Hellman–Feynmann theorem can be used to calculate forces acting on the nuclei within the DFT approximation:
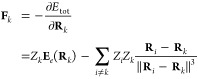
2where **E**_*e*_ is the electronic electric field and *Z*_*k*_ is the proton number of atom *k*.

### 2.2 Finite Nucleus Model

When multiwavelets are used to solve the DFT equations numerically, evaluating the electron–nucleus interaction integrals becomes challenging due to the singularity in the 1/*r* potential. In that case it is more efficient to use an approximate, smooth nuclear potential.

Harrison et al.^27^ and Yanai et al.^40^ propose the use of a smoothed nuclear potential of the form *U*(*r*) = *u*(*r*/*c*)/*c,* where
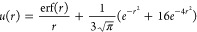
3*U*(*r*) is the smoothed nuclear potential with the property lim_*c*→0_*U*(*r*) = 1/*r*. Harrison et al.^27,40^ also provide a method to estimate the length scale *c* of the smoothed potential *U* to ensure overall precision in the calculations.

The introduction of the approximate nuclear potential *U* changes the first term in eq 2:
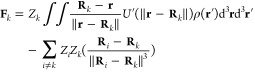
4The second term technically also changes, but the difference between *U*(*r*) and 1/*r* is insignificant when *r* is of the order of a bond length.

### 2.3 Stress Tensor

The stress tensor **σ**_*ij*_(**r**) is a symmetric tensor field whose divergence gives the force density:
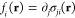
5The Einstein summation convention is used in eq 5 and will be applied throughout the rest of the paper. A useful property of the stress tensor is that it can be used to calculate the force **F** acting on a body located in the region of space **V** by integrating over the surface *S*(**V**) of **V**:
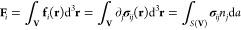
6Here, *n*_*j*_ is the *j*-th component of the normal vector of *S*(**V**). In the last step, the divergence theorem was used.

The total stress ⟨**σ**_*ij*_ ⟩ is an important observable in materials science. It can be used in bulk simulations to determine the optimal lattice shape or drive variable cell shape molecular dynamics simulations. The total stress represents the reaction of a system to a strain deformation *r*_*i*_*′* = (δ_*ij*_ + ε_*i j*_) *r*_*j*_, where ε_*ij*_ is the symmetric strain tensor. The total stress is ,^46,47^ where **R***′* contains the strained atomic positions and *E* is the potential energy of the system.

## 3 Method

In the Born–Oppenheimer approximation, the positions of the nuclei are fixed while the electrons occupy the lowest energy state. Due to the variational principle, there is no net force density acting on the electrons at any point **r**.^41^ By choosing a surface that encloses only a single nucleus and integrating the quantum mechanical stress tensor over this surface, it is possible to compute the forces acting on individual nuclei, using eq 6.^41^ This procedure can then be carried out for all the nuclei.

The quantum mechanical stress tensor density is the sum of the kinetic stress density , the Maxwell stress density  and the exchange-correlation stress density .^41,42,50,51^
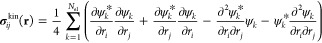
7
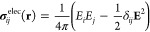
8
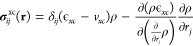
9Here, **E** is the sum of the electric field from the electrons ∫[ρ((**r** – **r***′*)/∥**r** – **r***′*∥^3^](**r** – **r***′*)*d*^3^**r***′* and the electric field from the nuclei . The second term in eq 9 accounts for the dependence of ϵ_xc_(ρ, ∇ρ) on the gradient of the charge density in the generalized gradient approximation.^46^

If the orbitals in eq 7 are real, the equation can be simplified further. Computing the second derivatives of the wave functions is computationally expensive. Eq 7 can be rewritten using the product rule such that the second derivatives act on the charge density ρ
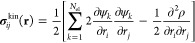
10The expectation value of the stress is given by −∑_*k*_⟨ψ_*k*_|*p*_*i*_*p*_*j*_|ψ_*k*_⟩.^41^ Interestingly, the quantum mechanical kinetic stress density is not given by −∑_*k*_ψ_*k*_*p*_*i*_*p*_*j*_ψ_*k*_ but by eq 7 or eq 10. A detailed derivation of the quantum mechanical kinetic stress density is presented by Maranganti and Sharma.^51^

## 4 Computational Details

As shown in eq 6, the force acting on atom *i* can be calculated by evaluating a surface integral over the quantum mechanical stress tensor where the surface encloses atom *i* and does not contain any other atom. The quantum mechanical stress density can be computed by adding eqs 7 to 9.

**Figure 1:**
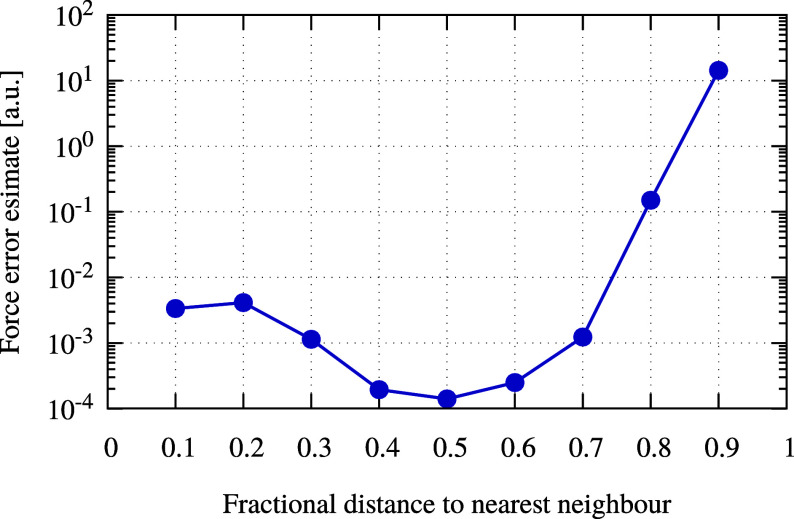
Force error estimate presented in eq 11 as a function of the radius of the integrations sphere for a water molecule.

To evaluate the integral in eq 6, one must define an appropriate surface containing a single atom. Since the stress tensor is integrated numerically, it is desirable that the integration domain is as smooth as possible such that the integrand varies slowly on this surface. A natural choice that fulfills both conditions is an atom-centered sphere with a radius of half the distance to the nearest neighbor of a given nucleus. This also minimizes possible interferences with the Cartesian adaptive grid used for the representation of functions with MWs. A force error estimate as a function of the radius of the integration sphere is shown in Figure 1. Another advantage of this choice is that it allows the use of Lebedev integration grids^52^ which give highly accurate results using only a few hundred integration points. Since the terms needed to compute the stress density are already part of a standard DFT calculation, calculating forces via surface integrals is highly efficient and typically requires only a fraction of the computational time of a single self-consistent field (SCF) iteration.

The force acting on atom *i* can be computed using the following ways:

1. Determine the distance *d*_*i*_ to the nearest neighbor of atom *i*. *d*_*i*_ defines the atom-centered integration surface.
2. Use the tabulated Lebedev integration points **x**_*k*_ and weights ω_*k*_ to generate the integration grid.
3. Sum up eqs 7 to 9 at all integration points **x**_*k*_ to get the stress density σ_*ij*_(**x**_*k*_)
4. The integral in eq 6 can be evaluated numerically using the Lebedev quatrature rule *F*_*i*_ = ∑_*k*_*w*_*k*_σ_*ij*_(**x**_*k*_)*n*_*j*_(**x**_*k*_). *n*_*j*_(**x**_*k*_) is the *j*-th component of the normal vector on the integration sphere at the point *x*_*k*_ which has the value *n*_*j*_(**x**_*k*_) = ((**R**_*i*_ – **x**_*k*_)/*d*_*i*_)_*j*_.

## 5 Results and Discussion

### 5.1 Accuracy of the Forces

**Figure 2:**
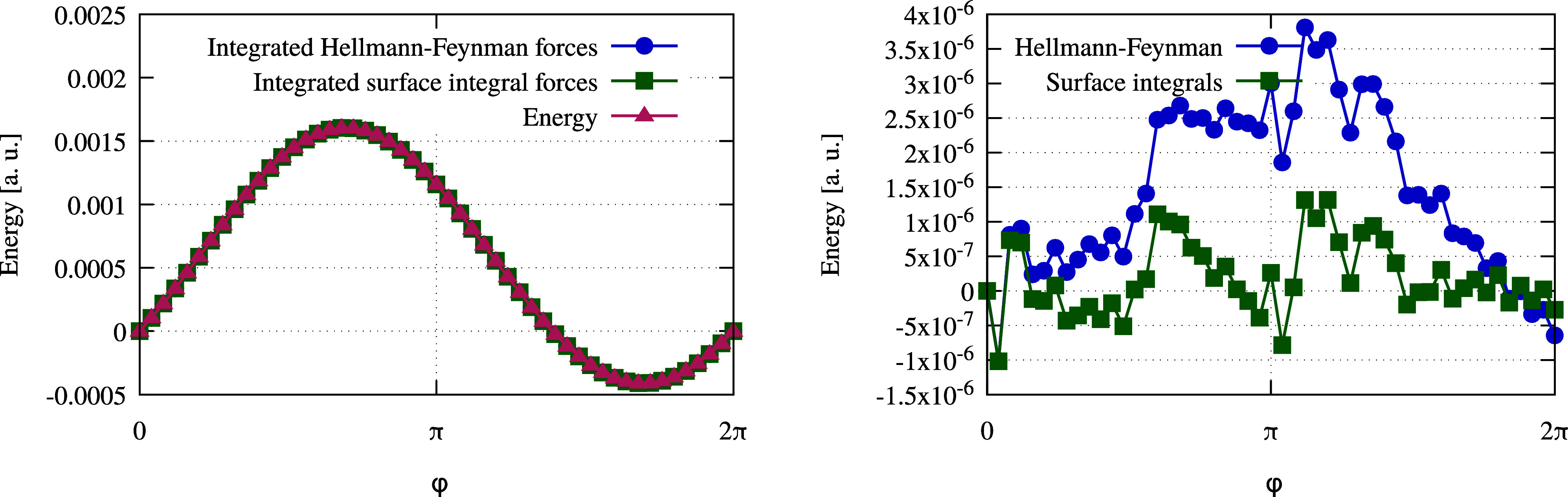
Line integration test to check the accuracy of the forces calculated with the Hellmann–Feynman theorem and with the surface integration method presented in this manuscript for a methanol molecule. Details of the test setup are explained in Section 5.1. On the left, we plot energies along a high dimensional circle, computed directly (red triangles) and as a result of the integration  for the Hellmann–Feynman (blue squares) and for the stress tensor (green circles) method. In the right figure, the difference between the energy along the circle and the corresponding line integral is shown. The smaller difference obtained with the stress tensor method (green circles) indicates that the forces are more consistent with the calculated DFT energy than for the Hellmann–Feynman method (blue squares). Energies were calculated using MRChem with a global precision of 10^–5^ and the local density approximation with the SVWN5 parametrization.^48,49^

An important property of forces is their consistency with the corresponding energies. Because the forces are just the negative gradient of the potential energy surface with respect to the atomic positions, that consistency can be checked using line integrals as displayed in Figure 2:

1. Generate a circle in the 3*N*_at_ dimensional configurational space that contains the atomic positions. Let φ ∈ (0, 2π] be the polar angle that parametrizes all points on that circle.
2. Consider the function *E*(φ) which is the potential energy of a point on that circle and  where **t̂**(φ) is the unit tangent vector at the point ϕ. If the forces are the exact gradient of the potential energy surface, those two functions are the same.
3. Evaluate *E*(φ) on a uniform grid and use these values to evaluate the integral numerically and compare the two functions in a plot.

In Figure 2, the results of the test are shown for a methanol molecule. Energies and forces were calculated with MRChem and a global precision of 10^–5^ in this comparison. The difference between the integrated forces and the exact energy is significantly smaller for the new method to calculate forces compared to the previous approach. This demonstrates, that the forces computed with surface integrals over the stress tensor are more accurate compared to the previous method. That test was repeated for multiple molecules with the same result.

Another useful measure to estimate the accuracy of the forces is presented by Gubler et al.^4^ where one uses the fact the sum of all forces equals zero. However, in a computer simulation, this relation is never exactly fulfilled. Let *s*_*i*_ (*i* = 1, 2, 3) be the sum of all *x*, *y,* and *z* components of the forces, respectively. Then, the standard deviation σ of the error in the numerically computed forces can be estimated using
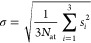
11This has been done for all the forces that were computed during the benchmark geometry optimizations. The average standard deviation for the forces computed using the Hellmann–Feynman theorem and the forces obtained with surface integrals over the stress tensor is shown in the last row of Table 1. The average standard deviation of the force error of forces calculated using surface integrals is almost ten times smaller than forces computed with the Hellmann–Feynman theorem.

**Table 1:** Force Error Measure from Eq. 11(4) for 22 Test Molecules^a^

	Hellman–Feynman	surface integrals
H_3_Al	1.1 × 10^–4^	2.3 × 10^–5^
H_2_Be	5.4 × 10^–6^	3.0 × 10^–5^
H_3_B	3.5 × 10^–5^	3.0 × 10^–5^
C_2_H_6_O	1.0 × 10^–4^	2.3 × 10^–5^
H_2_O	1.1 × 10^–4^	1.2 × 10^–5^
H_2_S	1.6 × 10^–4^	3.7 × 10^–5^
HCl	9.7 × 10^–5^	7.6 × 10^–5^
hexane	2.9 × 10^–5^	9.3 × 10^–6^
HF	2.8 × 10^–5^	3.2 × 10^–5^
HLi	2.8 × 10^–4^	1.2 × 10^–6^
methane	1.9 × 10^–5^	1.1 × 10^–5^
CH_4_O	6.2 × 10^–5^	9.6 × 10^–6^
H_2_Mg	9.6 × 10^–5^	4.7 × 10^–6^
HNa	4.9 × 10^–5^	4.1 × 10^–5^
H_3_N	4.6 × 10^–5^	4.8 × 10^–6^
H_3_P	2.0 × 10^–4^	7.4 × 10^–5^
H_4_Si	9.5 × 10^–5^	8.7 × 10^–6^
fluoropropylbenzene	4.4 × 10^–4^	4.2 × 10^–5^
hexanaldehyde	2.0 × 10^–4^	5.6 × 10^–5^
hexasilane	9.5 × 10^–5^	2.2 × 10^–5^
naphtalene	1.4 × 10^–4^	2.5 × 10^–5^
nitrobenzene	4.0 × 10^–4^	2.1 × 10^–5^
average	1.3 × 10^–4^	2.7 × 10^–5^

a The last line contains the average force error.

### 5.2 Geometry Optimizations

We conducted geometry optimizations for a diverse set of molecules with MRChem(17) where the new method presented in this article to calculate forces was implemented according to Section 4. In order to test the most general case, it was made sure that no atoms were on dyadic (coordinates that have the form *m*/2^*n*^ where *n* and *m* are integers) points. All the geometry optimizations were done at a global precision of 10^–5^ and a geometry optimization was considered converged when all force components were smaller than 10^–4^ a.u. The stabilized quasi-Newton method^3,4^ was used to conduct all geometry optimizations.

Geometry optimizations were conducted with forces using the new method presented in this study. In all the systems shown in Table 1, geometry optimizations using the new forces converged up to a force norm of 10^–4^ a.u. This is not the case when the forces are calculated using the Hellmann–Feynman theorem. In Table 1, the variance of the error in the forces is shown for the 22 test molecules. It is approximately 1 order of magnitude lower when forces are computed using surface integrals.

**Figure 3:**
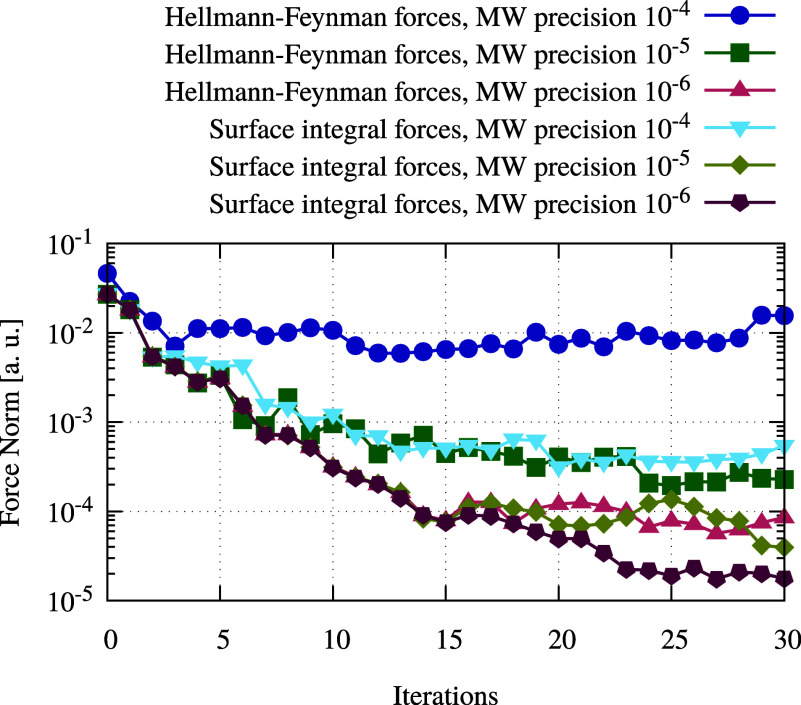
Convergence of the geometry optimizations of an ethanol molecule for the two methods to compute forces using MRChem and the BLYP functional, with various precision thresholds. The stress tensor method is able to achieve a reduction of the residual force norm of an order of magnitude for the same choice of precision parameter, compared to the Hellmann–Feynman method.

In Figure 3, the force norm is plotted against the number of geometry optimization iterations. When the forces were calculated using the Hellmann–Feynman method, the force norm stagnates 2 orders of magnitude before the MW precision. When the newly developed surface integral method is applied, the force norm improves by approximately 1 order of magnitude. Stagnation occurs when the force norm reaches about 1 order of magnitude above the MW precision. The error in the forces is a result of the error propagation from the SCF procedure using MWs. This shows that the surface integral method leads to a sizable reduction in error propagation.

### 5.3 Comparison with Gaussian Orbitals

We compared the forces obtained using MWs, with those obtained using the PySCF^53^ code with the correlation-consistent polarized valence bases (cc-pVnZ),^54,55^ where n stands for the number of zeta functions used and the presence of the “aug” aug indicates that diffuse GTOs were included in the basis. Additionally, a comparison was conducted using polarization-consistent (PC)^56^ basis sets, which are specifically optimized for DFT calculations. The presence of “aug” indicates once again that diffuse GTOs were included in the basis and “unc” indicates that the uncontracted version of the basis set was used. This is mainly done to verify our results and not as a comparative benchmark of forces obtained using various basis sets.

**Figure 4:**
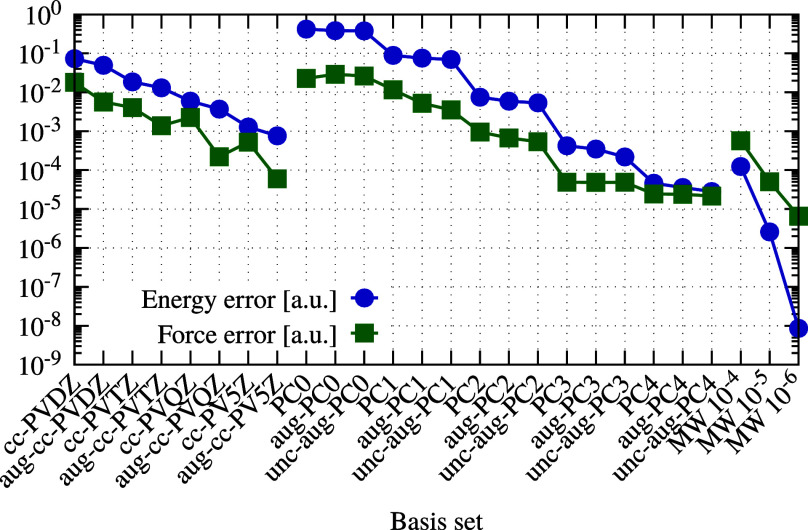
Convergence of Gaussian basis sets compared to MW results of a methanol molecule. For the MW reference calculation, a global precision of 10^–8^ a.u. was used and the forces were calculated using surface integrals over the stress tensor.

Methanol was chosen as a test molecule, and the Becke, Lee, Yang, and Parr (BLYP)^57,58^ exchange-correlation functional was employed. In Figure 4, for sufficiently large basis sets, the forces from Gaussian orbitals and MWs were consistent with the difference between MW and GTO forces falling below 10^–4^ a.u.

For the PC basis set, increasing the number of valence functions improves precision more effectively than augmenting or uncontracting the basis set. Notably, the condition number of the overlap matrix for the aug-PC4 and unc-aug-PC4 basis sets exceeds 10^8^, highlighting the limitations in the number of basis functions that GTOs can handle effectively.

Table 2 displays the time needed to compute the energy and the force for a methanol molecule using the BLYP^57,58^ exchange-correlation functional, and the Psi4^59^ electronic structure code. While calculations using small GTO basis sets are faster than MW calculations, exactly the opposite is the case when large GTO basis sets are used and the MW calculations are faster than GTOs. This demonstrates that MW is a great alternative to large Gaussian basis sets.

**Table 2:** Timings for Different Basis Sets for the Energy and Force Calculation of a Methanol Molecule and the BLYP Functional on a Machine with a 11th Generation Intel i7 CPU (11700) and 32 GB of RAM^a^

basis set	time (s)
10^–4^ (MW)	117
10^–5^ (MW)	322
10^–6^ (MW)	841
PC3	46
aug-PC3	174
PC4	543
aug-PC4	2349
cc-pVQZ	24
aug-cc-pVQZ	1712
cc-pV5Z	351
aug-cc-pV5Z	1817

a MW results were obtained using MRChem and GTO calculations performed with the Psi4^59^ code.

Combining the rate of convergence in Figure 4 to the complete basis set (CBS) limit with the timings presented in Table 2, it can be seen that to gain an order of magnitude in precision (one additional correct digit), an MW calculation becomes 2–3 times longer (e.g., from 117 s for MW4 to 322 s for MW5), whereas a similar gain in precision for GTOs requires at least a factor ten in computational cost (e.g., from 46 s for PC-3 to 543 s for PC-4 or from 24 s for cc-pVQZ to 351 s for cc-pV5Z). This is in line with previous results on energies^28,30,31^ and properties.^33,34^

We acknowledge that the results in this subsection do not constitute a comprehensive benchmark and only serve the purpose of demonstrating the correctness and effectiveness of the approach presented. A more comprehensive benchmark will be the focus of a future publication.

### 5.4 Machine-Learned Multiwavelet Potential Energy Surfaces

In recent years, machine-learned potentials (MLPs) have seen rapid development, maturing into a robust data-driven approach for representing potential energy surfaces. Several MLPs are now commonly used, such as Behler-Parrinello potentials,^47,60,61^ Neural Equivariant Interatomic Potentials (NeqIP),^62^ and Gaussian Approximation Potentials (GAP). These methods have demonstrated their ability to reliably learn both the potential energy surface and its derivatives the forces, making them powerful tools in computational chemistry and materials science.

MLPs typically consist of highly flexible model functions with numerous free parameters designed to predict the quantum mechanical energy of a molecular system. The forces are obtained by taking the negative analytic gradient of this model. To train the model for a specific system, these free parameters are adjusted based on reference data. It is crucial that the forces in this reference data are consistent with the energy since the model assumes this consistency when representing the potential energy surface.

To assess whether the forces obtained using the surface integral method are accurate enough for use as reference data in MLPs, a potential using the second-generation Behler–Parinello architecture was trained for the silane molecule. In order to generate reference data, a molecular dynamics simulation was performed using the FHI-aims^12^ code with a time step of 1 fs and a temperature of 500 K. After 50,000 steps, every tenth structure from the last 40,000 steps was added to the data set, resulting in 4000 structures. These were randomly divided into training (1800 structures), test (200 structures), and validation (2000 structures) sets. The MLP was optimized using the training set, with the test set used to monitor performance and avoid overfitting. The validation set was not used in any way during the training phase and was only used after the training phase to validate the accuracy of the model.

**Figure 5:**
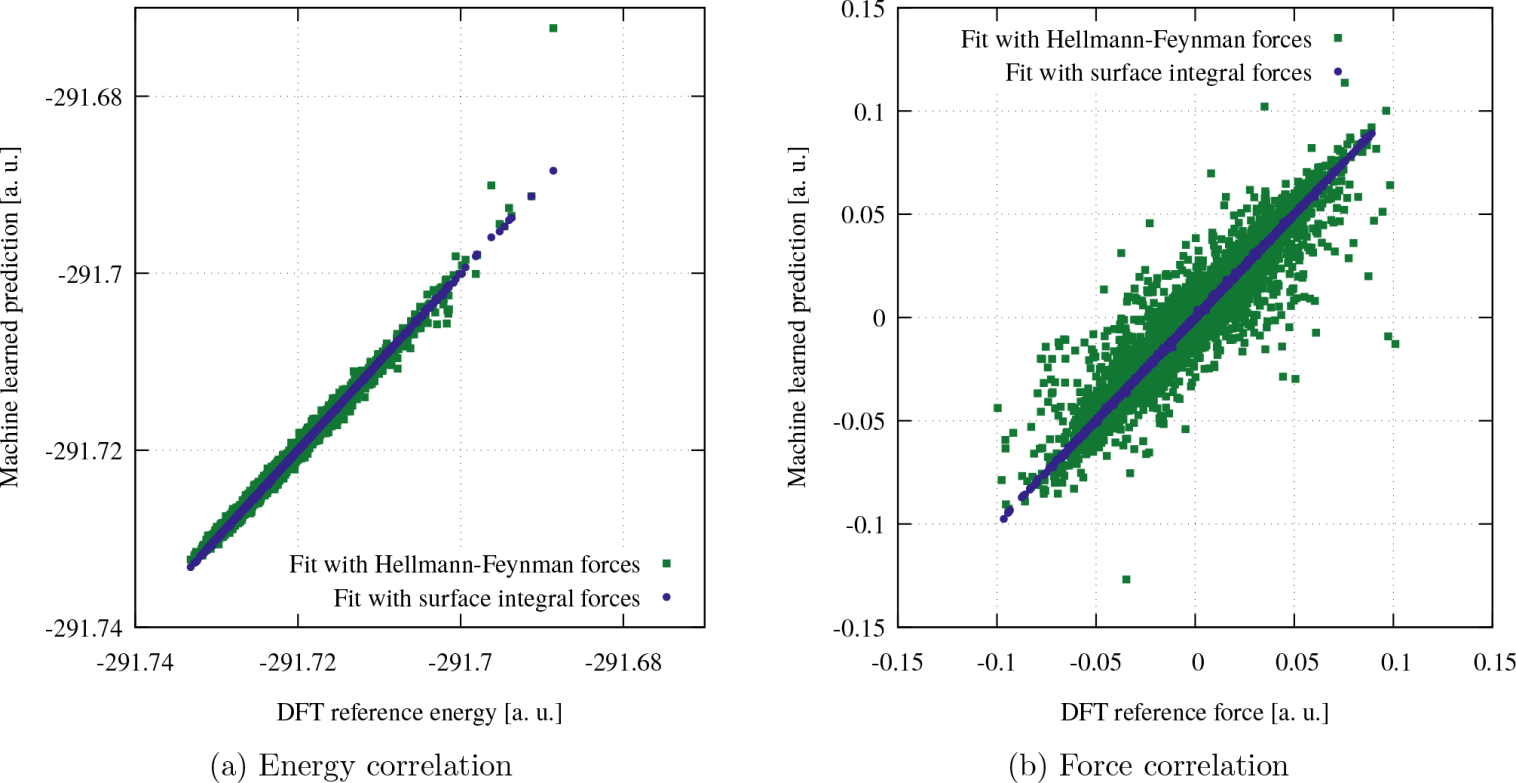
Correlation of the machine learned energies (left) and forces (right) against the correct values obtained with DFT for both MLPs.

Energies and forces for the training data were calculated using MWs and MRChem with a global wavelet precision of 10^–4^ a.u. Two sets of forces were computed: one using the Hellmann–Feynman method and another using the surface integral method. Correspondingly, two neural network potentials were trained using each set of forces. The training was carried out using the RuNNeR code.^63,64^ For both methods to compute forces, a machine-learned potential was trained using exactly the same parameters, training, test, and validation sets. In Figure 5, the correlation between the predicted energies and forces and the corresponding DFT values is illustrated for the validation set.

Compared with the reference values of the validation set, we find that the accuracy of the machine-learned surface integral forces is significantly better than the accuracy of the Hellmann–Feynman forces. This further demonstrates the superiority of the method presented in this manuscript. The higher quality of the forces computed with surface integrals also affects the energy correlation as seen in Figure 5a. Since the forces are the negative analytic gradient of the machine-learned model with respect to the atomic positions, energies can not be trained accurately when the forces are not accurate enough which is the case for the Hellmann–Feynman forces. For the MLP trained using Hellmann–Feynman forces, the root-mean-square error (RMSE) of the validation set is 7.7 × 10^–4^ a.u. for the energies and 5.0 × 10^–3^ a.u. for the forces. In contrast, the MLP trained with surface integral forces achieves an accuracy that is an order of magnitude higher with an RMSE of 4.7 × 10^–5^ a.u. for the energies and 1.6 × 10^–4^ a.u. for the forces. Notably, the RMSE for the surface integral-based MLP approaches the MW precision used in the DFT calculation which was 10^–4^ a.u.

### 5.5 Molecular Dynamics

In molecular dynamics simulations, precise force calculations are essential to ensure the conservation of total energy. To evaluate whether the precision of surface integral-based forces is sufficient to achieve this, a water molecule was used as a test system. The nuclei were propagated using the velocity Verlet algorithm^6,65^ with a time step of 10.3 au (0.25 fs). Initial velocities were sampled from a Boltzmann distribution at a temperature of 500 K.

Two molecular dynamics simulations were performed starting from the same initial geometry and velocities. One simulation employed Hellmann–Feynman-based forces, while the other utilized surface integral-based forces. Energies and forces were computed with a MW precision of 10^–5^.

**Figure 6:**
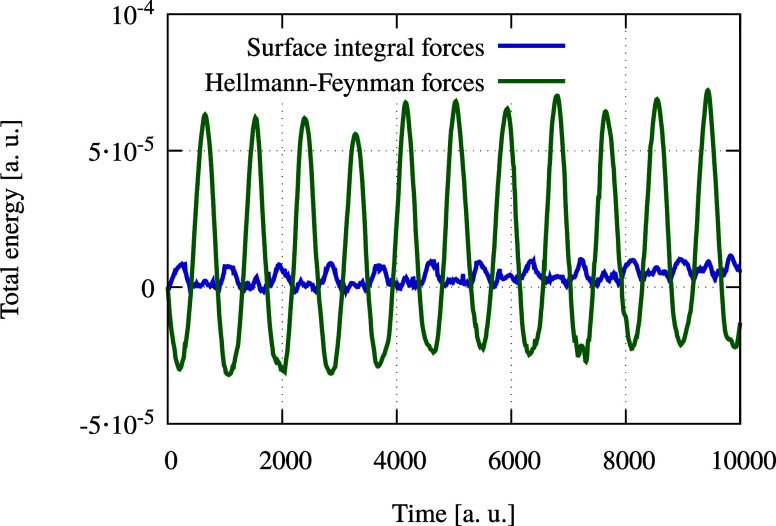
Total energy against time of a water molecule in a molecular dynamics simulation.

The total energy over time is shown in Figure 6, demonstrating that simulations using surface integral-based forces exhibit smaller fluctuations in total energy. Both methods achieve long-term energy conservation, with no significant energy drift observed in either case.

A similar comparison was conducted with a MW precision of 10^–4^, where neither method achieved satisfactory energy conservation.

## 6 Conclusions

We have introduced an alternative method for computing forces within the framework of density functional theory, which is particularly suited for scenarios where multiresolution wavelets are employed to represent orbitals and densities. Through extensive benchmarking, we compared our method against the current state-of-the-art technique for force computation. Our results indicate that using the Hellman–Feynman theorem to calculate forces with MWs is around an order of magnitude less precise than our newly proposed method that uses surface integrals. The current implementation is limited to semilocal functionals within the generalized gradient approximation (GGA) approximation, but work is underway to extend this approach to include exact exchange and metaGGA functionals.

We also performed a line integration test to assess the consistency of the computed forces with the potential energy, and a test to estimate the force error. Our method demonstrated superior accuracy in both tests, significantly outperforming the current approach.

To corroborate the precision of MW calculations, energies and forces were compared with those obtained using Gaussian basis sets. The results demonstrate excellent agreement when large Gaussian basis sets are employed.

These findings clearly illustrate that computing forces using surface integrals over the quantum mechanical stress tensor offers substantial advantages over the traditional method based on the Hellmann–Feynman theorem. Our approach not only improves the accuracy of force computations but also enhances the overall efficiency of geometry optimization procedures in DFT calculations.

The comparison of machine-learned potentials in Section 5.4 demonstrates that, unlike the previous method for computing forces with MWs, the surface integral-based approach is precise enough to serve as training data for machine-learned potentials. This enables the creation of machine-learned potentials free of basis set errors, using MRChem to generate highly precise reference data, making it possible to create machine-learned potentials with a previously unattainable level of precision.

## Accession Codes

A reference implementation of the method presented in this paper can be found in the following GitHub repository, and will soon be included in the next MRChem release: https://github.com/moritzgubler/mrchem/tree/surface:forces

## References

[ref1] Hohenberg P.; Kohn W. Inhomogeneous Electron Gas. Phys. Rev. 1964, 136, B864–B871. 10.1103/PhysRev.136.B864.

[ref2] Kohn W.; Sham L. J. Self-Consistent Equations Including Exchange and Correlation Effects. Phys. Rev. 1965, 140, A1133–A1138. 10.1103/PhysRev.140.A1133.

[ref3] Schaefer B.; Alireza Ghasemi S.; Roy S.; Goedecker S. Stabilized quasi-Newton optimization of noisy potential energy surfaces. J. Chem. Phys. 2015, 142, 034112 10.1063/1.4905665. 25612694

[ref4] Gubler M.; Krummenacher M.; Huber H.; Goedecker S. Efficient variable cell shape geometry optimization. Journal of Computational Physics: X 2023, 17, 100131 10.1016/j.jcpx.2023.100131.

[ref5] Krummenacher M.; Gubler M.; Finkler J. A.; Huber H.; Sommer-Jörgensen M.; Goedecker S. Performing highly efficient Minima Hopping structure predictions using the Atomic Simulation Environment (ASE). SoftwareX 2024, 25, 101632 10.1016/j.softx.2024.101632.

[ref6] Verlet L. Computer ”Experiments” on Classical Fluids. I. Thermodynamical Properties of Lennard-Jones Molecules. Phys. Rev. 1967, 159, 98–103. 10.1103/PhysRev.159.98.

[ref7] Feynman R. P. Forces in Molecules. Phys. Rev. 1939, 56, 340–343. 10.1103/PhysRev.56.340.

[ref8] Boys S. F.; Egerton A. C. Electronic wave functions - I. A general method of calculation for the stationary states of any molecular system. Proceedings of the Royal Society of London. Series A. Mathematical and Physical Sciences 1950, 200, 542–554.

[ref9] Eschrig H.; Bergert I. An optimized LCAO version for band structure calculations application to copper. physica status solidi (b) 1978, 90, 621–628. 10.1002/pssb.2220900221.

[ref10] Averill F. W.; Ellis D. E. An efficient numerical multicenter basis set for molecular orbital calculations: Application to FeCl4. J. Chem. Phys. 1973, 59, 6412–6418. 10.1063/1.1680020.

[ref11] Zunger A.; Freeman A. J. Self-consistent numerical-basis-set linear-combination-of-atomic-orbitals model for the study of solids in the local density formalism. Phys. Rev. B 1977, 15, 4716–4737. 10.1103/PhysRevB.15.4716.

[ref12] Blum V.; Gehrke R.; Hanke F.; Havu P.; Havu V.; Ren X.; Reuter K.; Scheffler M. Ab initio molecular simulations with numeric atom-centered orbitals. Comput. Phys. Commun. 2009, 180, 2175–2196. 10.1016/j.cpc.2009.06.022.

[ref13] Pulay P. Ab initio calculation of force constants and equilibrium geometries in polyatomic molecules. Mol. Phys. 1969, 17, 197–204. 10.1080/00268976900100941.

[ref14] Pathak S.; López I. E.; Lee A. J.; Bricker W. P.; Fernández R. L.; Lehtola S.; Rackers J. A. Accurate Hellmann–Feynman forces from density functional calculations with augmented Gaussian basis sets. J. Chem. Phys. 2023, 158, 014104 10.1063/5.0130668. 36610956

[ref15] Genovese L.; Videau B.; Ospici M.; Deutsch T.; Goedecker S.; Méhaut J.-F. Daubechies wavelets for high performance electronic structure calculations: The BigDFT project. Comptes Rendus. M é canique 2011, 339, 149–164. 10.1016/j.crme.2010.12.003.

[ref16] Harrison R. J.; et al. MADNESS: A Multiresolution, Adaptive Numerical Environment for Scientific Simulation. SIAM Journal on Scientific Computing 2016, 38, S123–S142. 10.1137/15M1026171.

[ref17] Bast R.; Bjo̷rgve M.; Brakestad A.; Di Remigio R.; FredianiA L.; Gerez G.; Jensen S. R.; Wind P. MRChem: MultiResolution Chemistry. 2022.

[ref18] Goedecker S.; Teter M.; Hutter J. Separable dual-space Gaussian pseudopotentials. Phys. Rev. B 1996, 54, 1703–1710. 10.1103/PhysRevB.54.1703. 9986014

[ref19] Hartwigsen C.; Goedecker S.; Hutter J. Relativistic separable dual-space Gaussian pseudopotentials from H to Rn. Phys. Rev. B 1998, 58, 3641–3662. 10.1103/PhysRevB.58.3641. 9986014

[ref20] Gygi F. All-Electron Plane-Wave Electronic Structure Calculations. J. Chem. Theory Comput. 2023, 19, 1300–1309. 10.1021/acs.jctc.2c01191. 36757291 PMC9979607

[ref21] Lehtola S. Accuracy of a Recent Regularized Nuclear Potential. J. Chem. Theory Comput. 2023, 19, 4033–4039. 10.1021/acs.jctc.3c00530. 37354116 PMC10339670

[ref22] Jensen S. R.; Saha S.; Flores-Livas J. A.; Huhn W.; Blum V.; Goedecker S.; FredianiC L. The Elephant in the Room of Density Functional Theory Calculations. J. Phys. Chem. Lett. 2017, 8, 1449–1457. 10.1021/acs.jpclett.7b00255. 28291362

[ref23] Alpert B.; Beylkin G.; Gines D.; Vozovoi L. Adaptive Solution of Partial Differential Equations in Multiwavelet Bases. J. Comput. Phys. 2002, 182, 149–190. 10.1006/jcph.2002.7160.

[ref24] Alpert B. K. A Class of Bases in $L^2$ for the Sparse Representation of Integral Operators. SIAM Journal on Mathematical Analysis 1993, 24, 246–262. 10.1137/0524016.

[ref25] Beylkin G.; Monzón L. On approximation of functions by exponential sums. Applied and Computational Harmonic Analysis 2005, 19, 17–48. 10.1016/j.acha.2005.01.003.

[ref26] Yanai T.; Fann G. I.; Gan Z.; Harrison R. J.; Beylkin G. Multiresolution quantum chemistry in multiwavelet bases: Hartree–Fock exchange. J. Chem. Phys. 2004, 121, 6680–6688. 10.1063/1.1790931. 15473723

[ref27] Harrison R. J.; Fann G. I.; Yanai T.; Gan Z.; Beylkin G. Multiresolution quantum chemistry: Basic theory and initial applications. J. Chem. Phys. 2004, 121, 11587–11598. 10.1063/1.1791051. 15634124

[ref28] Jensen S. R.; Saha S.; Flores-Livas J. A.; Huhn W.; Blum V.; Goedecker S.; FredianiE L. The Elephant in the Room of Density Functional Theory Calculations. J. Phys. Chem. Lett. 2017, 8, 1449–1457. 10.1021/acs.jpclett.7b00255. 28291362

[ref29] Yanai T.; Harrison R. J.; Handy N. C. Multiresolution quantum chemistry in multiwavelet bases: time-dependent density functional theory with asymptotically corrected potentials in local density and generalized gradient approximations. Mol. Phys. 2005, 103, 413–424. 10.1080/00268970412331319236.

[ref30] Jensen S. R.; Eikås R. D. R.; FredianiG L.; Wind P.; Bjo̷rgve M.; Brakestad A.; Gabriel A. Gerez S. MRChem Multiresolution Analysis Code for Molecular Electronic Structure Calculations: Performance and Scaling Properties. J. Chem. Theory Comput. 2023, 19, 137–146. 10.1021/acs.jctc.2c00982. 36410396 PMC9835826

[ref31] Brakestad A.; Wind P.; Jensen S. R.; FredianiH L.; Hopmann K. H. Multiwavelets applied to metal–ligand interactions: Energies free from basis set errors. J. Chem. Phys. 2021, 154, 214302 10.1063/5.0046023. 34240981

[ref32] Hurtado A.; Sekino H.; Harrison R. J. Benchmarking Correlation-Consistent Basis Sets for Frequency-Dependent Polarizabilities with Multiresolution Analysis. J. Chem. Theory Comput. 2024, 20, 5145–5156. 10.1021/acs.jctc.4c00394. 38842252 PMC11209939

[ref33] Jensen S. R.; Flå T.; Jonsson D.; Monstad R. S.; Ruud K.; FredianiF L. Magnetic properties with multiwavelets and DFT: the complete basis set limit achieved. Phys. Chem. Chem. Phys. 2016, 18, 21145–21161. 10.1039/C6CP01294A. 27087397

[ref34] Brakestad A.; Jensen S. R.; Wind P.; D’Alessandro M.; Genovese L.; Hopmann K. H.; Frediani L. Static Polarizabilities at the Basis Set Limit: A Benchmark of 124 Species. J. Chem. Theory Comput. 2020, 16, 4874–4882. 10.1021/acs.jctc.0c00128. 32544327 PMC7467643

[ref35] Valeev E. F.; Harrison R. J.; Holmes A. A.; Peterson C. C.; Penchoff D. A. Direct Determination of Optimal Real-Space Orbitals for Correlated Electronic Structure of Molecules. J. Chem. Theory Comput. 2023, 19, 7230–7241. 10.1021/acs.jctc.3c0073. 37791808

[ref36] Bischoff F. A.; Valeev E. F. Computing molecular correlation energies with guaranteed precision. J. Chem. Phys. 2013, 139, 114106 10.1063/1.4820404. 24070278

[ref37] Brakestad A.; Jensen S. R.; Tantardini C.; Pitteloud Q.; Wind P.; Užulis J.; Gulans A.; Hopmann K. H.; FredianiI L. Scalar Relativistic Effects with Multiwavelets: Implementation and Benchmark. J. Chem. Theory Comput. 2024, 20, 728–737. 10.1021/acs.jctc.3c01095. 38181377 PMC10809714

[ref38] Tantardini C.; Di Remigio Eikås R.; Bjo̷rgve M.; Jensen S. R.; Frediani L. Full Breit Hamiltonian in the Multiwavelets Framework. J. Chem. Theory Comput. 2024, 20, 882–890. 10.1021/acs.jctc.3c01056. 38163290 PMC10809419

[ref39] Anderson J.; Sundahl B.; Harrison R.; Beylkin G. Dirac-Fock calculations on molecules in an adaptive multiwavelet basis. J. Chem. Phys. 2019, 151, 234112 10.1063/1.5128908. 31864249

[ref40] Yanai T.; Fann G. I.; Gan Z.; Harrison R. J.; Beylkin G. Multiresolution quantum chemistry in multiwavelet bases: Analytic derivatives for Hartree–Fock and density functional theory. J. Chem. Phys. 2004, 121, 2866–2876. 10.1063/1.1768161. 15291596

[ref41] Nielsen O. H.; Martin R. M. Quantum-mechanical theory of stress and force. Phys. Rev. B 1985, 32, 3780–3791. 10.1103/PhysRevB.32.3780. 9937528

[ref42] Nielsen O. H.; Martin R. M. First-Principles Calculation of Stress. Phys. Rev. Lett. 1983, 50, 697–700. 10.1103/PhysRevLett.50.697.

[ref43] Baer R.; Neuhauser D.; Rabani E. Self-Averaging Stochastic Kohn-Sham Density-Functional Theory. Phys. Rev. Lett. 2013, 111, 106402 10.1103/PhysRevLett.111.106402. 25166686

[ref44] Lin L.; Lu J.; Ying L.; E W. Adaptive local basis set for Kohn–Sham density functional theory in a discontinuous Galerkin framework I: Total energy calculation. J. Comput. Phys. 2012, 231, 2140–2154. 10.1016/j.jcp.2011.11.032.

[ref45] Zhang G.; Lin L.; Hu W.; Yang C.; Pask J. E. Adaptive local basis set for Kohn–Sham density functional theory in a discontinuous Galerkin framework II: Force, vibration, and molecular dynamics calculations. J. Comput. Phys. 2017, 335, 426–443. 10.1016/j.jcp.2016.12.052.

[ref46] Dal Corso A.; Resta R. Density-functional theory of macroscopic stress: Gradient-corrected calculations for crystalline Se. Phys. Rev. B 1994, 50, 4327–4331. 10.1103/PhysRevB.50.4327. 9976731

[ref47] Gubler M.; Finkler J. A.; Schäfer M. R.; Behler J.; Goedecker S. Accelerating Fourth-Generation Machine Learning Potentials Using Quasi-Linear Scaling Particle Mesh Charge Equilibration. J. Chem. Theory Comput. 2024, 20, 7264–7271. 10.1021/acs.jctc.4c00334. 39151921 PMC11360134

[ref48] Slater J. C. A Simplification of the Hartree-Fock Method. Phys. Rev. 1951, 81, 385–390. 10.1103/PhysRev.81.385.

[ref49] Vosko S. H.; Wilk L.; Nusair M. Accurate spin-dependent electron liquid correlation energies for local spin density calculations: a critical analysis. Can. J. Phys. 1980, 58, 1200–1211. 10.1139/p80-159.

[ref50] Nielsen O. H.; Martin R. M. Stresses in semiconductors: Ab initio calculations on Si, Ge, and GaAs. Phys. Rev. B 1985, 32, 3792–3805. 10.1103/PhysRevB.32.3792. 9937529

[ref51] Maranganti R.; Sharma P. Revisiting quantum notions of stress. Proceedings of the Royal Society A: Mathematical, Physical and Engineering Sciences 2010, 466, 2097–2116. 10.1098/rspa.2009.0636.

[ref52] Lebedev V. I. Spherical quadrature formulas exact to orders 25–29. Siberian Mathematical Journal 1977, 18, 99–107. 10.1007/BF00966954.

[ref53] Sun Q.; Berkelbach T. C.; Blunt N. S.; Booth G. H.; Guo S.; Li Z.; Liu J.; McClain J. D.; Sayfutyarova E. R.; Sharma S.; Wouters S.; Chan G. K.-L. PySCF: the Python-based simulations of chemistry framework. WIREs Computational Molecular Science 2018, 8, e1340 10.1002/wcms.1340.

[ref54] Dunning J.; Thom H. Gaussian basis sets for use in correlated molecular calculations. I. The atoms boron through neon and hydrogen. J. Chem. Phys. 1989, 90, 1007–1023. 10.1063/1.456153.

[ref55] Nagy B.; Jensen F. Reviews in Computational Chemistry. Reviews in Computational Chemistry 2017, 30, 93–149. 10.1002/9781119356059.ch3.

[ref56] Jensen F. Polarization consistent basis sets: Principles. J. Chem. Phys. 2001, 115, 9113–9125. 10.1063/1.1413524.

[ref57] Becke A. D. Correlation energy of an inhomogeneous electron gas: A coordinate-space model. J. Chem. Phys. 1988, 88, 1053–1062. 10.1063/1.454274.

[ref58] Lee C.; Yang W.; Parr R. G. Development of the Colle-Salvetti correlation-energy formula into a functional of the electron density. Phys. Rev. B 1988, 37, 785–789. 10.1103/PhysRevB.37.785. 9944570

[ref59] Smith D. G. A.; et al. PSI4 1.4: Open-source software for high-throughput quantum chemistry. J. Chem. Phys. 2020, 152, 184108 10.1063/5.0006002. 32414239 PMC7228781

[ref60] Behler J.; Parrinello M. Generalized Neural-Network Representation of High-Dimensional Potential-Energy Surfaces. Phys. Rev. Lett. 2007, 98, 146401 10.1103/PhysRevLett.98.146401. 17501293

[ref61] Ko T. W.; Finkler J. A.; Goedecker S.; Behler J. A fourth-generation high-dimensional neural network potential with accurate electrostatics including non-local charge transfer. Nat. Commun. 2021, 12, 398 10.1038/s41467-020-20427-2. 33452239 PMC7811002

[ref62] Batzner S.; Musaelian A.; Sun L.; Geiger M.; Mailoa J. P.; Kornbluth M.; Molinari N.; Smidt T. E.; Kozinsky B. E(3)-equivariant graph neural networks for data-efficient and accurate interatomic potentials. Nat. Commun. 2022, 13, 2453 10.1038/s41467-022-29939-5. 35508450 PMC9068614

[ref63] Behler J. Constructing High-Dimensional Neural Network Potentials: A Tutorial Review. Int. J. Quantum Chem. 2015, 115, 1032–1050. 10.1002/qua.24890.

[ref64] Behler J. First Principles Neural Network Potentials for Reactive Simulations of Large Molecular and Condensed Systems. Angew. Chem., Int. Ed. 2017, 56, 12828–12840. 10.1002/anie.201703114. 28520235

[ref65] Swope W. C.; Andersen H. C.; Berens P. H.; Wilson K. R. A computer simulation method for the calculation of equilibrium constants for the formation of physical clusters of molecules: Application to small water clusters. J. Chem. Phys. 1982, 76, 637–649. 10.1063/1.442716.

